# We Are One: Multispecies Metabolism of a Biofilm Consortium and Their Treatment Strategies

**DOI:** 10.3389/fmicb.2021.635432

**Published:** 2021-01-28

**Authors:** Ruchika Vinod Joshi, Cindy Gunawan, Riti Mann

**Affiliations:** ^1^iThree Institute, University of Technology Sydney, Sydney, NSW, Australia; ^2^School of Chemical Engineering, University of New South Wales, Sydney, NSW, Australia

**Keywords:** biofilms, multispecies, metabolism, treatment, interactions

## Abstract

The ecological and medical significance of bacterial biofilms have been well recognized. Biofilms are harder to control than their planktonic free-living counterparts and quite recently, the focus of the study has shifted to the multispecies consortia, which represent the vast majority of real-case infection scenarios. Studies have begun to explore the complex interspecies interactions within these biofilms. However, only little attention is currently given to the role of cellular metabolites in the cell-to-cell communication. The concentration gradients of metabolic substrates and products affect the spatial growth of bacteria in multispecies biofilm. This, if looked into more deeply, can lead to identification of potential therapies targeting the specific metabolites and hence the coordinated protection in the bacterial community. Herein, we review the interspecies communications, including their metabolic cross-talking, in multispecies biofilm, to signify the importance of such interactions on the initial formation and subsequent growth of these biofilms. Multispecies biofilms with their species heterogeneity are more resilient to antimicrobial agents than their single species biofilm counterparts and this characteristic is of particular interest when dealing with pathogenic bacteria. In this Review, we also discuss the treatment options available, to include current and emerging avenues to combat pathogenic multispecies biofilms in the clinical, environmental, as well as industrial settings.

## Introduction

Bacteria typically live in complex biological communities, known as biofilms; which dominate all habitats on the surface of the Earth, except the oceans, where 20–80% of bacterial cells exist as biofilms (Hall et al., 2014; Flemming and Wuertz, 2019). Biofilms are often comprised of multiple microbial species, each carrying its own unique features, imparting certain evolved and unique functions that are not present in their mono-species counterparts (Flemming et al., 2016). Such biofilms, referred to as the multispecies biofilms, are commonly found on a wide range of medical devices and are associated with a significant amount of human bacterial infections, posing a serious human health concern and economic burden to the health-care systems (Bryers, 2008; Hall et al., 2014; Kvich et al., 2020).

The formation of multispecies biofilms is a complex process, coordinated by the sequential interaction of different species. These interactions in the bacterial community are highly specific and often change the structural and functional dynamics of the whole biofilm community, enhancing protection as well as virulence characteristics (Yang et al., 2011). These spatial interactions, arising from a high level of species heterogeneity in these biofilms, renders these biofilms highly resilient to conventional antimicrobial treatments, urging the need for effective alternative therapies (Flemming et al., 2016). Understanding the interspecies communications in multispecies biofilms will enable the discovery of novel targets for controlling biofilms in the environmental, industrial and clinical settings. Herein, we discuss important recent literatures to showcase our current understanding of the interspecies interactions in a multispecies biofilm. Later in the review, we describe the metabolic heterogeneity in such biofilms, a factor influencing their antibiotic susceptibility; and finally, we highlight the recent advancements in the treatment of biofilm-related infections, centering more on the discovery of non-antibiotic alternative treatment options.

## Building the Multispecies Biofilms

Critical to the formation and development of multispecies biofilms is the cell-to-cell interactions, termed as co-adhesion and co-aggregation, which together foster mutualistic communications between adjacent cells in a biofilm. The adherence of bacterial cells to immobilized cells is called as co-adhesion whereas the binding of microbial cells in suspension is known as co-aggregation (Kolenbrander et al., 2010). These two binding interactions provide diverse attachment sites for the planktonic bacteria to adhere to in the process of biofilm development (Foster and Kolenbrander, 2004). The formation of multispecies biofilms is a complex process that in general is categorized into three steps: (1) the attachment of primary colonizers to the surface, their clonal growth and the production of exopolysaccharides, protein adhesins, amyloids and nucleic acids, which together form the EPS (Extracellular Polymeric Substance); resulting in the formation of microcolonies, (2) the attachment of secondary colonizers to these microcolonies, followed by their proliferation, and (3) dispersion of biofilm, mediated by environmental stimulus, which allows the cells to detach and establish a new biofilm at other sites (Hobley et al., 2015; Steinberg and Kolodkin-Gal, 2015; Salinas et al., 2020). The first step, dependent on the bacterial physiochemical interactions, is highly specific; such that the primary colonizers can only co-aggregate with other primary colonizers and not with any secondary colonizing bacteria. The co-adhesion of initial primary colonizers is crucial for the biofilm colonization, whereas an increase in EPS production is essential for the attachment of secondary colonizers to the microcolonies, as EPS works as an intercellular-cement in biofilm proliferation by sticking the cells together and mediating a successive co-aggregation as the biofilm matures (Rickard et al., 2003).

## Interspecies Interactions in Multispecies Biofilms

**FIGURE 1:**
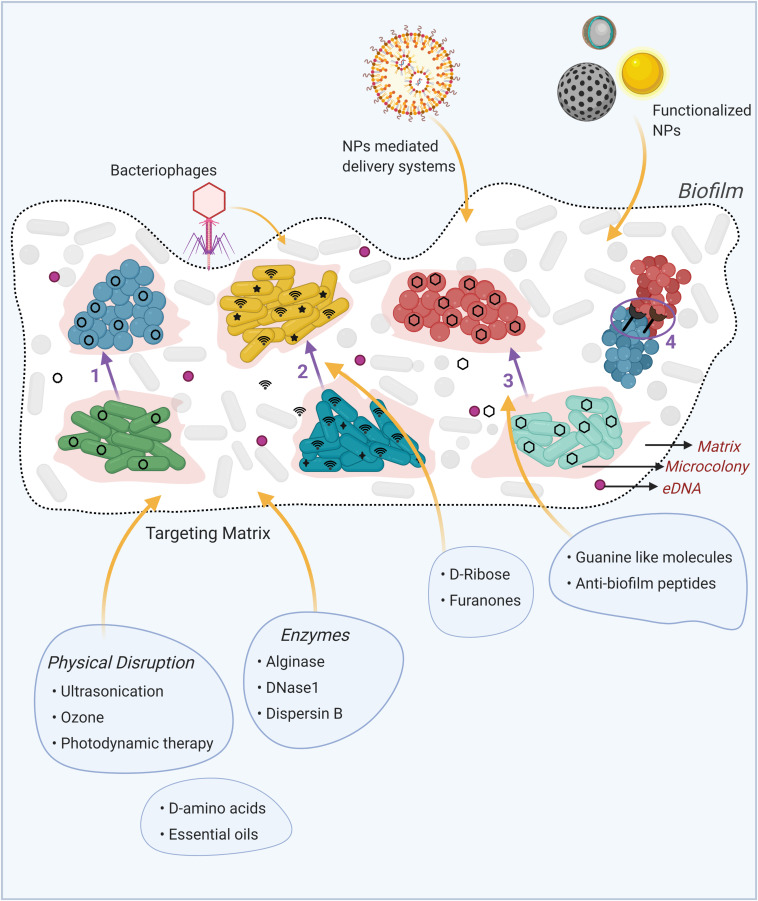
Schematic representation of the mechanisms of interspecies interactions in multispecies biofilms and innovative biofilm-therapeutic strategies. **(1)** Horizontal gene transfer via plasmid conjugation, where plasmid (black circles) is transferred from one species (green cells) to another (blue cells). **(2)** Quorum sensing through intraspecific (black stars) and interspecific (wifi signals) communication by diffusible molecules. **(3)** Metabolic cooperation where the by-product of one species (green cells) serve as nutrient (black hexagons) for another species (red cells). **(4)** Physical interactions, where specific cell-to-cell interactions occurs between cells of different species through specific cell surface receptors. Available treatment options to combat biofilms are depicted in illustrations around the biofilm, with yellow arrows pointing to their target in the biofilm. NPs: Nanoparticles, eDNA: extracellular DNA. Created using Biorender.

The interspecies interactions within a biofilm have been a recent focus of many studies. Bacteria in a multispecies biofilm consortium mainly communicate via four highly specific mechanisms, namely, the physical interactions, exchange of genetic material, metabolic networking and by using diffusible signals, which in many cases, only take place when the respective bacterial species form a multispecies biofilm (Blehert et al., 2003; Flemming et al., 2016) (Figure 1). These interspecies communications, depending on the intricate molecular mechanisms, can cause social behaviors that can be neutral, cooperative or competitive for the species involved (Burmølle et al., 2014), with the latter two types mainly shaping the organization and functionality of a multispecies biofilm community.

Cooperation within the biofilm community is facilitated through synergistic interactions that modulate the differential gene expression and cellular responses of each species, allowing them to evolve and better adapt to the biofilm conditions. One such example is the association between *Pseudomonas putida* KT2440 and *Acinetobacter* sp. C6, wherein *P. putida* evolves in the presence of *Acinetobacter* by altering its outer core lipopolysaccharide synthesis. This results in the formation of rough variants that show enhanced fitness by acquiring more benzoate – a by-product of *Acinetobacter*, making the overall community more stable and productive (Hansen et al., 2007). Synergistic interactions can also result from transfer of genetic material between different species, either through plasmid conjugation or DNA transformation, providing stability to the biofilm and helps in resisting attacks from phages, antibiotics and toxins (Wang et al., 2002; Molin and Tolker-Nielsen, 2003; Reisner et al., 2006). For instance, the biofilm-stimulating effects were observed due to the conjugative transfer of F-like and IncIα plasmids between genetically diverse strains of *Escherichia coli* (Reisner et al., 2006). Cell-to-cell physical interactions are also an important factor in the synergistic interactions in biofilms, resulting in the formation of cellular aggregates. This, for example, has been seen in multispecies biofilms causing dental plaque in oral cavities (Kolenbrander et al., 2010). The inter-cellular communication, a key process in the formation of oral biofilms, between *Actinomyces naeslundii* and *Streptococcus oralis* [via the universal intergeneric signaling molecule – Autoinducer 2 (AI2)], only occurs when these bacteria co-aggregate (Hardie and Heurlier, 2008). Research inquiries have indicated that synergistic interactions cause a particular bacterium to thrive better in the presence of other bacteria than they would on their own. For instance, *Bacillus cereus* is known to release thiazolyl peptide antibiotics – thiocillins, which increase the population of matrix-producing cells of *Bacillus subtilis*, thereby enhancing its biofilm forming properties (Bleich et al., 2015). Synergistic interactions could also manifest in the form of an enhanced growth rate when the species are present together, as demonstrated by a threefold increase in multispecies biofilm of four soil isolates: *Stenotrophomonas rhizophila*, *Xanthomonas retroflexus*, *Microbacterium oxydans*, and *Paenibacillus amylolyticus*, compared to their respective single species biofilms. This synergistic effect is suggested to result from their shared evolutionary history that facilitates nutrient cross-feeding between them (Ren et al., 2014).

Competitive interactions result from antagonistic relationships in a biofilm, whereby one bacterial species produces molecules that inhibit the growth of other species. This has been observed in the dual species biofilm of *Lactobacilli* and *Streptococcus*, in which the biofilm forming ability of *Streptococcus* on glass surfaces was inhibited by *Lactobacilli* in a pH-dependent manner (Söderling et al., 2011). In other case, *Pseudomonas aeruginosa* displays a “blanketing” effect on *Agrobacterium tumefaciens* microcolonies, when grown as dual biofilms, facilitating better growth of the former bacterium. This effect is thought to result from the motile nature of *P. aeruginosa* cells, as the flagellar and type IV pili mutants of the bacterium did not exhibit the “blanketing” effect (An et al., 2006). Antagonistic relationship is also observed in the dual biofilm of *P. aeruginosa* and *Candida albicans*, in which *P. aeruginosa* restricts the maturation of *C. albicans* biofilms by regulating the expression of adhesion molecules, quorum sensing (QS) molecules and the virulence genes (Holcombe et al., 2010). In the meat processing industry, *Salmonella* biofilms are shown to be inhibited by *P. aeruginosa* through the production of acyl-homoserine lactone (AHL), which is hypothesized to modulate the cell division in *Salmonella*, also affecting the chemical composition of EPS, reducing the adhesion ability of *Salmonella* (Wang et al., 2013). Antagonistic activity of several molecules released by the soil-bacterium *B. subtilis* has been demonstrated against a range of different bacterial genera. This includes; surfactin, which is shown to arrest the development of aerial hyphae in *Streptomyces coelicolor* (Straight and Joanne, 2006), chlorotetain, which degrades the colonies of *Staphylococcus epidermidis* when these two bacteria come in proximity on the human skin (Hernandez-Valdes et al., 2020), surfactin and plipastatin, which alters the virulence factors of *Staphylococcus aureus* (Gonzalez et al., 2011), and surfactin and cannibalism toxin, both of which eliminate the colonies of *Bacillus simplex* and *Bacillus toyonensis* (Rosenberg et al., 2016). On the other hand, research enquiries have also identified the antagonistic effect of compounds released by other bacterial species on the growth, physiology and biofilm formation of *B. subtilis*. For example, the active compound 2,4-diacetylphloroglucinol (DAPG), secreted by *Pseudomonas protegens*, is shown to cause phenotypic alterations and inhibit biofilm formation in *B. subtilis* (Powers et al., 2015), and linearmycins, a family of polyketides, produced by the soil bacterium *Streptomyces* sp. has been seen to cause cellular lysis of *B. subtilis* (Stubbendieck and Straight, 2015).

Communication through chemical signaling, referred to as QS, plays an important role in the establishment of multispecies biofilms. QS systems in *P. aeruginosa*, a strong biofilm former, are highly complex and among the most studied systems. The production of amino-4-methoxy-trans-3-butenoic acid, a QS-regulated toxic compound produced by *P. aeruginosa*, has been associated with inhibition of other pathogenic microbes (Rojas Murcia et al., 2015). The dual biofilm of *Streptococcus mitis* and *P. aeruginosa* are commonly found in the endotracheal tubes of infants. Although *S. mitis* is not a pathogen by itself, it releases the QS autoinducer-2 (AI-2) molecule, which aids the growth of *P. aeruginosa*, enhancing its biofilm forming capability and apparently, its pathogenicity (Wang et al., 2016). QS has a decisive role to play in the pathogenicity of *P. aeruginosa*, as indicated by the differential QS profiling of its clinical isolates and lab-cultured strains, primarily arising from the relative abundance of a QS molecule AHL (Singh et al., 2000).

In addition to the specific molecular mechanisms and physical interactions discussed above, metabolic communication also facilitates inter-species cross-talk in a biofilm. These metabolic interactions, dealt in the next section of this review, play important roles in spatial organization of microbes and a proper functioning of a biofilm.

## Metabolic Communications in Multispecies Biofilms

Matrix production, in addition to establishing the biofilm structure, also results in metabolic diversification by controlling the physical interactions between bacterial cells and their immediate environment. This enables metabolic cross-feeding, promoting the development of metabolically differentiated subpopulations in a biofilm and making the biofilms a metabolically heterogeneous community [refer to the recent reviews by Evans et al. (2020) and Povolotsky et al. (2021) for a comprehensive discussion on metabolic heterogeneity in biofilms]. Interspecies interactions, along with the biofilm structure, influence the signals that promote metabolic differentiation, eventually shaping the nutrient and chemical gradient of a biofilm. Interspecies interactions facilitate metabolic cooperation in a biofilm when the metabolic by-products of one species are used as nutrients by the other species (Christensen et al., 2002). One example is the use of lactic acid from *S. oralis* by *Veillonella* sp. in the oral biofilm formed by these two species (Periasamy and Kolenbrander, 2010). Structurally, EPS helps in the absorption of nutrients, creating a nutrient gradient, whereby, by-products of one species can be used as nutrient by the other species, reducing unwanted, toxic waste in biofilms (Elias and Banin, 2012). The spatial organization and composition of *P. protegens*, *P. aeruginosa*, and *Klebsiella pneumoniae* multispecies biofilm is influenced by nutrient availability, which has an effect on their survival under stressful conditions (Lee et al., 2014). Metabolically distinct subzones, based on oxygen availability, were observed in *P. aeruginosa* PA14 biofilms; whereby cells in anoxic regions produced lactate by expressing lactate dehydrogenase (LdhA). The lactate was then cross-fed to cells in the oxic conditions, activating the expression of *lldE*, the gene that encodes for lactate oxidizing enzyme – lactate dehydrogenase, involved in utilization of lactate (Lin et al., 2018). This metabolic cross-feeding allows the use of a carbon source – lactate, which would else persist as a toxic metabolic waste product within the biofilm. Similarly, metabolic cross-feeding mediated by high redox potential compounds – phenazines, has been observed in between the oxic and anoxic regions of a *P. aeruginosa* biofilm. Phenazines, produced in oxic regions of the biofilm, were observed to migrate to oxygen-limited regions, where they served as alternate electron acceptors, supporting the metabolic activity in these zones (Williamson et al., 2012; Schiessl et al., 2019).

As the metabolic state of a cell is the determinant of its antibiotic susceptibility (Stokes et al., 2019), numerous studies have looked into the metabolic status of individual cells as well as the metabolic cross-feeding in multicellular systems (Evans et al., 2020); however similar research on biofilms is still scanty, and only a few metabolites involved have been identified. Biofilm formation in various bacterial species is related to an increase in the activity of cyclic diguanylate monophosphate (c-di-GMP) (Ross et al., 1987). In Gram-negative bacteria, this secondary messenger molecule regulates biofilm formation by acting as the main switch between sessile and motile mode of bacterial growth, enabling attachment of cells on surfaces through a signaling cascade (Simm et al., 2004). In *P. aeruginosa*, c-di-GMP regulates the production of exopolysaccharide alginate, a major component of the biofilm matrix and is a factor in the persistence of *P. aeruginosa* biofilms, frequently seen in lung infections (Römling and Balsalobre, 2012). An elevated level of c-di-GMP is also noted in the rough small colony variants (RSCV) of *P. aeruginosa*, a hyper biofilm former, showing an increased tolerance to antimicrobials (Starkey et al., 2009). *S. aureus* produces c-di-AMP (cyclic diadenosine monophosphate) as a secondary messenger, instead of c-di-GMP, which produces components, most likely adhesins, required for biofilm formation (Corrigan et al., 2011).

Cyclic adenosine monophosphate (cAMP) is another important secondary messenger molecule that has been shown to affect biofilm formation process through multiple signal transduction cascades (Jackson et al., 2002; McDonough and Rodriguez, 2012; Kalivoda et al., 2013). In *Vibrio cholerae*, it activates biofilm formation by negatively regulating the biofilm repressor HapR (QS transcriptional regulator) and positively regulating the biofilm activator VpsR (transcriptional regulator of the *Vibrio* polysaccharide synthesis operon) (Liang et al., 2007). It also acts as a biofilm repressor by negatively regulating an activator – diguanylate cyclase CdgA of biofilm formation (Fong and Yildiz, 2008). cAMP was also found to inhibit EPS synthesis and the formation of a multilayer biofilm (Houot and Watnick, 2008). Another metabolite, ppGpp plays crucial role in the formation and maintenance of biofilms, as the ppGpp mutants were observed to form loose biofilms due to their decreased ability to adhere to a surface (De la Fuente-Núñez et al., 2014). Another study found that eliminating ppGpp synthesis in a biofilm, reduced bacterial growth compared to the wild type cells, and the cells that grew were tolerant to the DNA replication targeting antibiotic ofloxacin (Nguyen et al., 2011).

The interspecies interactions in a biofilm, described so far, enhance the survival of bacterial biofilms, which pose a significant issue in industrial and clinical settings (Kolenbrander and London, 1993; Galié et al., 2018). Hence, in the subsequent section, we discuss the recent technological advancements in controlling biofilms and identify potential interspecies interactions that can be targeted to combat a vast array of biofilm-related infections.

## Innovative Treatment Strategies for Controlling Biofilms

The complex biofilm matrix makes the biofilms resilient to almost all antimicrobial treatments. Besides the use of antibiotics, research work on novel biofilm eradication strategies have been primarily focused on the disruption of the protective EPS matrix, leading to biofilm disintegration. These approaches, considered an effective strategy to control biofilms, are schematically summarized in Figure 1. Targeting alginate, one of the major component of the EPS in *P. aeruginosa* biofilm, using the alginase enzyme, has been identified as a potential strategy for the treatment of cystic fibrosis patients (Glonti et al., 2010). Combination therapies, comprising of a matrix degrading agent and an antibacterial agent, have also shown efficacy in dealing with biofilm infections. An antibiotic – Dnase1 (degrades extracellular DNA) combination therapy was shown to disrupt the EPS, enhancing the antibiofilm effects of antibiotics in clearing bacterial single-species biofilms (Fanaei Pirlar et al., 2020). A combination of DNase1 and plant-based essential oils also disrupted the biofilm of methicillin-resistant *S. aureus* (Rubini et al., 2018). Using Dispersin B, a biofilm-dispersing enzyme, in combination with peptides, eradicated ∼70% of *S. epidermidis* biofilms compared to only ∼35% by Dispersin B alone (Chen and Lee, 2018). Further, D-amino acids from *B. subtilis*, known to signal for biofilm disassembly, were found to inhibit the development of *S. aureus* biofilms (Chen et al., 2020). Intriguingly, honey – a natural product, has also shown anti-biofilm effects by inhibiting *P. aeruginosa* biofilm formation and reducing its established biofilms (Lu et al., 2019). The cell-free supernatant of the yeast *Saccharomyces cerevisiae* has been shown to exhibit anti-biofilm effects on *Listeria monocytogenes* biofilms, primarily by decreasing the EPS production (Kim et al., 2021). Another innovative approach in treatment of biofilm-related infections is the use of iron chelators, which have shown significant anti-biofilm activity on both Gram-positive and Gram-negative bacteria (Richter et al., 2017).

Other treatment strategy involves the development of nanoparticle (NP)-based systems to target biofilms. Proteinase K-capped gold NPs were shown to degrade the mature biofilms of *P. fluorescens* by disrupting its EPS components (Habimana et al., 2018), while silver (Ag) NPs at concentrations as low as 1μg/mL have shown efficacy in inhibiting the formation of *P. aeruginosa* biofilms (Kora and Arunachalam, 2011). In addition to their use as anti-biofilm agents, nanosystems have also been successfully applied as carriers to enhance antibiotic delivery in biofilm systems by co-mobilizing a matrix-disrupting agent and an antibacterial agent onto NPs (Baelo et al., 2015; Tan et al., 2018, 2020).

Studies have shown the use of QS inhibitors in the treatment of biofilm-forming pathogenic infections. In the dual biofilm of *S. mitis* and *P. aeruginosa*, whereby the AI-2 molecule released from *S. mitis* promotes the pathogenicity of *P. aeruginosa*; D-ribose, has been shown to inhibit the activity of AI-2 by competing for its receptor site (Wang et al., 2016). Naturally occurring halogenated molecules, the furanones, can inhibit the QS signaling molecule “AHL,” resulting in reduced biofilm thickness and swarming motility of *E. coli*, *V. cholerae*, and *P. aeruginosa* (Proctor et al., 2020). Bacteriophages have also shown promising results in treatment of highly antibiotic-resistant biofilm infections, as they prevented *Klebsiella* biofilm formation on urinary catheters and demonstrated significant clinical improvements in chronic otitis patients (caused by *P. aeruginosa* and *S. aureus* biofilms) (Wright et al., 2009; Townsend et al., 2020). The use of physical techniques for biofilm dispersal has also gained attention in the last decade. Combination ultrasonication-ozone treatment, for example, has been shown to eliminate *L. monocytogenes* biofilms from stainless steel surfaces through disruption of proteins in the EPS (Baumann et al., 2009; Yu et al., 2020). Photodynamic therapy, using the photosensitizing molecule 5-aminolevulinic acid was able to inactivate cells in mono-species antibiotic-resistant *S. aureus* and *S. epidermidis* biofilms (Li et al., 2013).

The cellular metabolites involved in interspecies interactions in multispecies biofilms can be a potential target options for the treatment of the biofilms, for example, c-di-GMP, a signaling molecule required for biofilm formation is the prime target candidate. A recent *in silico* study identified “guanine-like” molecules that could limit diguanylate cyclase activity, leading to reduced intracellular c-di-GMP signals, which in turn, inhibited the initial attachment and induced dispersion of *P. aeruginosa* biofilm (Sambanthamoorthy et al., 2014). Anti-biofilm peptides, a subset of host defense peptides, have been shown to interact with and degrade the ppGpp molecule, which plays a role in biofilm establishment. As low as 0.8 μg/mL concentration of the peptide was able to initiate dispersal of *P. aeruginosa* biofilms, while treatment at 10 μg/mL caused complete destruction of the biofilms (De la Fuente-Núñez et al., 2014). However, despite showing efficacy, these peptides are susceptible to degradation by the innate presence of bacterial proteases. To address this, studies have been developing d-enantiomeric protease resistant peptides, which show a 10-fold decreased biofilm inhibition concentration compared to the protease susceptible peptides (de la Fuente-Núñez et al., 2015).

Above suggested treatments are generally applicable for both single-species and multispecies biofilms. However, it is now well-established that during infections, bacteria are mostly found coexisting with other species, showing interspecies interactions, metabolic heterogeneity and cross-feeding; which all can enhance the cellular pathogenicity and antibiotic tolerance. Indeed, studies have identified that metabolites involved in interspecies interactions can interfere with ‘drug-cell’ interactions and metabolic differentiation does contribute to antibiotic tolerance in multicellular systems, which can critically influence our ability to treat infections (Co et al., 2019; Schiessl et al., 2019). Hence, the treatment outcome from a multispecies biofilm with complex cross-species interactions would be different from a monospecies biofilm, highlighting the need to incorporate these interactions while designing our treatment strategies.

## Conclusion

The negative effects of bacterial biofilms are well recognized. Many biofilm treatment strategies have been focused on targeting the protective polymer matrix that shields the bacterial community from antimicrobial agents. Apart from such structural targeting, we found from this review that the physiological cell-to-cell interactions in biofilm can indeed serve as another important avenue worth exploring as the potential treatment target. A number of physiological interactions, primarily the receptor-mediated cell aggregation, intercellular signaling, metabolic communication and horizontal gene transfer, are known to maintain a tightly regulated and functional biofilm biomass, for a community-associated protection against stress, including from antimicrobial agents. While inhibitors of intercellular signaling (QS inhibitors) have been identified, only little progress however, has been made on the targeting of other intercellular interaction pathways. As an example, the targeting of metabolic communications such as those via the global transcriptional regulators such as cyclic AMP or c-di-GMP, is anticipated to inhibit the cell-to-cell interactions, even those between different species, presenting a potential implication in the treatment of multispecies biofilms. The Review highlights the need to shift biofilm eradication strategies from the current targeting of biofilm structural entities to targeting metabolic communications that underlie the cell-to-cell interactions, which is anticipated to offer long-term treatment solutions.

## Author Contributions

RM conceived, revised, and edited the manuscript. RJ conducted the literature study. RJ and RM wrote the manuscript. CG provided feedback and edited the manuscript. All authors gave final approval of the version to be submitted and published.

## Conflict of Interest

The authors declare that the research was conducted in the absence of any commercial or financial relationships that could be construed as a potential conflict of interest.
